# A Novel Seven Gene Signature-Based Prognostic Model to Predict Distant Metastasis of Lymph Node-Negative Triple-Negative Breast Cancer

**DOI:** 10.3389/fonc.2021.746763

**Published:** 2021-09-16

**Authors:** Wenting Peng, Caijin Lin, Shanshan Jing, Guanhua Su, Xi Jin, Genhong Di, Zhiming Shao

**Affiliations:** ^1^Department of Breast Surgery, Fudan University Shanghai Cancer Center, Shanghai, China; ^2^Key Laboratory of Breast Cancer in Shanghai, Fudan University Shanghai Cancer Center, Shanghai, China; ^3^Department of Oncology, Shanghai Medical College, Fudan University, Shanghai, China; ^4^Department of Breast Surgery, The Affiliated Changzhou No. 2 People’s Hospital of Nanjing Medical University, Changzhou, China; ^5^Department of Nursing Administration, Fudan University Shanghai Cancer Center, Shanghai, China

**Keywords:** triple-negative breast cancer, distant metastasis, prognostic biomarker, modeling, transcriptomics

## Abstract

**Background:**

The prognosis of lymph node-negative triple-negative breast cancer (TNBC) is still worse than that of other subtypes despite adjuvant chemotherapy. Reliable prognostic biomarkers are required to identify lymph node-negative TNBC patients at a high risk of distant metastasis and optimize individual treatment.

**Methods:**

We analyzed the RNA sequencing data of primary tumor tissue and the clinicopathological data of 202 lymph node-negative TNBC patients. The cohort was randomly divided into training and validation sets. Least absolute shrinkage and selection operator Cox regression and multivariate Cox regression were used to construct the prognostic model.

**Results:**

A clinical prognostic model, seven-gene signature, and combined model were constructed using the training set and validated using the validation set. The seven-gene signature was established based on the genomic variables associated with distant metastasis after shrinkage correction. The difference in the risk of distant metastasis between the low- and high-risk groups was statistically significant using the seven-gene signature (training set: *P* < 0.001; validation set: *P* = 0.039). The combined model showed significance in the training set (*P* < 0.001) and trended toward significance in the validation set (*P* = 0.071). The seven-gene signature showed improved prognostic accuracy relative to the clinical signature in the training data (AUC value of 4-year ROC, 0.879 *vs.* 0.699, *P* = 0.046). Moreover, the composite clinical and gene signature also showed improved prognostic accuracy relative to the clinical signature (AUC value of 4-year ROC: 0.888 *vs.* 0.699, *P* = 0.029; AUC value of 5-year ROC: 0.882 *vs.* 0.693, *P* = 0.038). A nomogram model was constructed with the seven-gene signature, patient age, and tumor size.

**Conclusions:**

The proposed signature may improve the risk stratification of lymph node-negative TNBC patients. High-risk lymph node-negative TNBC patients may benefit from treatment escalation.

## Introduction

Breast cancer is estimated to be the most common cancer diagnosed in women and the second leading cause of cancer-related death in the United States in 2021 (1). Triple-negative breast cancer (TNBC) is characterized by a lack of expression of estrogen receptor (ER), progesterone receptor (PR), and human epidermal growth factor receptor 2 (HER2), representing 10%-20% of all breast cancers (2, 3). TNBC is more likely to show lymph node involvement at diagnosis and exhibit invasive and metastatic tendencies (2, 4). Nonetheless, the incidence of lymph node-negative TNBC has markedly increased owing to early detection and initiated screening programs (5–8).

To date, lymph node-negative TNBC is generally considered at moderate risk of disease recurrence and is often recommended for adjuvant chemotherapy (9). Small lymph node-negative tumors tend to have an excellent prognosis without chemotherapy (10). However, the risk of metastasis and death of partial lymph node-negative TNBC patients is still high despite the high proportion of adjuvant chemotherapy (2, 11, 12). A more quantitative approach is required to inform the risk of distant metastasis and individualized treatment in lymph node-negative TNBC.

Several multigene assays have been developed to facilitate prognosis prediction and treatment planning in early-stage breast cancer, but most of the enrolled patients are hormone receptor-positive (13–15). Although many publications have attempted to identify gene signatures that predict the prognosis of TNBC patients, several limitations need to be considered due to the limited sample size and incomplete follow-up information (16–20). Above all, most previous studies include all TNBC patients as a cohort. Because lymph node status is a well-known prognostic value, there is an urgent need to identify a robust risk stratification tool for lymph node-negative TNBC patients (21, 22). Based on detailed clinicopathological information, well-documented follow-up, and complete RNA-sequencing data, we constructed a gene expression-based prognostic signature combined with clinicopathological factors to provide quantitative predictions of short- and long-term disease outcomes for Chinese lymph node-negative TNBC patients.

## Materials and Methods

### Patient Samples and Study Design

We included 202 eligible patients from our previously published cohort of 465 primary TNBC patients treated at Fudan University Shanghai Cancer Center (FUSCCTNBC) (23). Patients were included based on the following criteria: histologic diagnosis of lymph node-negative TNBC with RNA-sequencing data and follow-up information for recurrence and metastasis. The RNA-sequencing data are available in the Sequence Read Archive (RNA-seq: SRP157974). Patients with contralateral breast cancer, lymph node recurrence, and unknown sites of recurrence were excluded. Lymph node status was independently confirmed by two experienced pathologists. The date of diagnosis of metastasis was defined when metastasis was either confirmed by biopsy or clinically diagnosed. The follow-up of this cohort was completed on June 11, 2019. Distant metastasis-free survival (DMFS) was defined as the interval between diagnosis and the first distant metastasis (viscera/bone/brain). Patients without events were censored from the time point of the last follow-up.

### Ethics Statement

The present study was reviewed and approved by the Ethics Committee of Fudan University Shanghai Cancer Center (Ethics number: 050432-4-1212B). The patients provided written informed consent to participate in this study.

### Gene Selection and Risk-Score Algorithm

To identify mRNAs of prognostic value, analysis for differentially expressed mRNAs between two groups was performed using the *limma* package (version 3.48.0) in R software. We also performed Gene Set Enrichment Analysis (GSEA) of differentially expressed genes between the two groups with or without distant metastasis using the RNA-sequencing data and GSEA software (GSEA_4.1.0) (24, 25).

The cohort was randomly divided into the training set (n=142) and validation set (n=60) at a ratio of 7 to 3 by the *caret* package (version 6.0-88) in R software. Pearson chi-square test or Fisher’s exact test was used to ensure that there was no significant difference and that no bias was introduced in clinicopathological characteristics between the two sets. Least absolute shrinkage and selection operator (LASSO) Cox regression analysis was performed to further filter the differentially expressed mRNAs. A multivariate Cox regression model was used to determine the coefficient of each factor. The risk score of each model was used to estimate the probability of distant metastasis. The genomic risk score was calculated from individual gene expression measurements as follows: Genomic risk score = (β*_B3GALT5-AS1_* × *B3GALT5-AS1*) + (β*_DNER_* × *DNER*) + (β*_CSN1S1_* × *CSN1S1*) + (β*_KIF5A_* × *KIF5A*) + (β*_SIX3_* × *SIX3*) + (β*_NOTUM_* × *NOTUM*) + (β*_CPS1_* × *CPS1*). The clinical risk score was calculated as follows: Clinical risk score = β_Age_ × Age (years)+ β_Tumor size_ × Tumor size (cm). The combined risk score was calculated as follows: Combined risk score = β_Gene score_ × Genomic risk score + β_Clinical score_ × Clinical risk score.

### Validation of Different Prognostic Models

Patients were stratified into high- and low-risk groups based on optimum cutoff risk scores determined by the “surv_cutpoint” function in the *survminer* package (version 0.4.9) in R software. Kaplan-Meier analyses and log-rank tests were performed to assess the differences in DMFS between the high- and low-risk groups. The time-dependent receiver operating characteristic (ROC) curve was used to measure the prognostic performance by comparing the area under the ROC curve (AUC) values.

### Construction and Validation of a Nomogram Model

Based on data availability and clinical evidence (9, 26, 27), a nomogram was constructed integrating the seven-gene risk score, age of the patients at surgery, and pathological tumor size. We measured the predictive accuracy of the nomogram *via* Harrell’s concordance index (C-index) in the training and validation sets. In addition, the predictive capacity of the nomogram was also evaluated using calibration curve and decision curve analysis (DCA).

### Statistical Analysis

Pearson’s chi-square test or Fisher’s exact test was used to compare the clinical and pathological characteristics between the training set and validation set. All statistical analyses were performed using the SPSS 22.0 (SPSS Inc.) or R software (version 4.1.0, www.r-project.com). A value of *P* < 0.05 was considered statistically significant.

## Results

### Patient Characteristics

The clinical and pathological characteristics of 202 patients and their primary tumors are summarized in **Table 1**. Of 202 lymph node-negative TNBC patients, the median follow-up was 68.2 months (interquartile range, 57.6-80.6 months). Overall, 12 (5.9%) cases with distant metastasis were observed. Of the 12 patients, 4 (33.3%) patients had multisite metastasis, and 7 (58.3%) patients died due to breast cancer during follow-up. The median tumor size and age of the patients at surgery in this study cohort were 2.5 centimeters (range 0.8-12.0) and 53 years (range 25-82), respectively.

**Table 1:** Clinicopathological characteristics of patients and their tumors.

Characteristics	Number of patients (%)			*P* ^a^
	Whole set	Training set	Validation set	
**Age, years**				0.865
≤50	86 (42.6%)	61 (43.0%)	25 (41.7%)	
>50	116 (57.4%)	81 (57.0%)	35 (58.3%)	
**Menopausal status**				0.468
Premenopausal	75 (37.1%)	55 (38.7%)	20 (33.3%)	
Postmenopausal	127 (62.9%)	87 (61.3%)	40 (66.7%)	
**Histological grade**				0.183
I	35 (17.3%)	27 (19.0%)	8 (13.3%)	
II	13 (6.4%)	9 (6.3%)	4 (6.7%)	
III	134 (66.3%)	96 (67.6%)	38 (63.3%)	
Unknown	20 (9.9%)	10 (7.0%)	10 (16.7%)	
**Tumor size**				0.239
≤2cm	85 (42.1%)	64 (45.1%)	21 (35.0%)	
>2-5cm	111 (55.0%)	75 (52.8%)	36 (60.0%)	
>5cm	6 (3.0%)	3 (2.1%)	3 (5%)	
**Ki-67**				0.820
≤20%	28 (13.9%)	20 (14.1%)	8 (13.3%)	
>20%	169 (83.7%)	119 (83.8%)	50 (83.3%)	
Unknown	5 (2.5%)	3 (2.1%)	2 (3.3%)	
**Chemotherapy**				0.644
No	6 (3.0%)	4 (2.8%)	2 (3.3%)	
Yes	188 (93.1%)	131 (92.3%)	57 (95.0%)	
Unknown	8 (4.0%)	7 (4.9%)	1 (1.7%)	
**Radiotherapy**				0.861
No	180 (89.1%)	127 (89.4%)	53 (88.3%)	
Yes	21 (10.4%)	14 (9.9%)	7 (11.7%)	
Unknown	1 (0.5%)	1 (0.7%)	0 (0.0%)	
**Metastasis**				0.345
No	190 (94.1%)	135 (95.1%)	55 (91.7%)	
Yes	12 (5.9%)	7 (4.9%)	5 (8.3%)	

a P values were calculated using Pearson’s chi-square test or Fisher’s exact test to compare the clinical and pathological characteristics between the training set and validation set.

### Construction and Validation of the Novel Seven-Gene Signature

**Figure 1:**
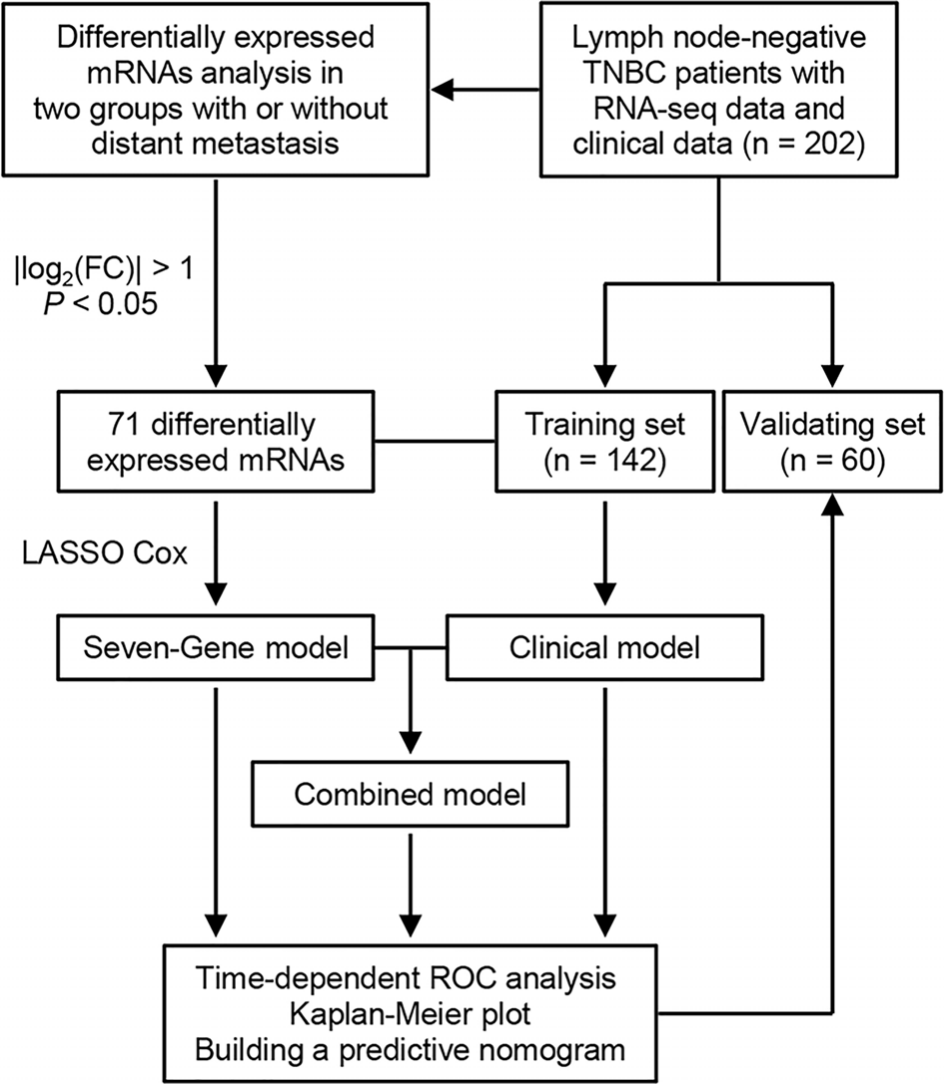
Flowchart of study design. TNBC, triple-negative breast cancer; FC, fold change; LASSO, least absolute shrinkage and selection operator; ROC, receiver operating characteristic.

**Figure 2:**
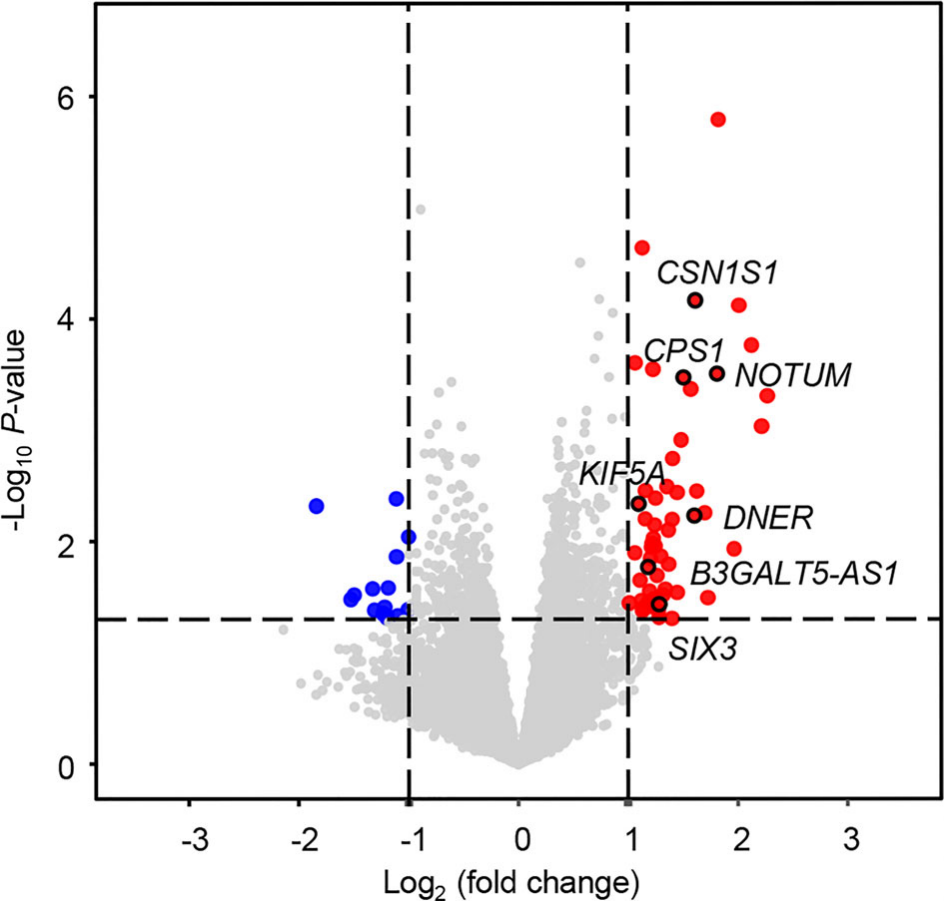
Volcano plot for differentially expressed mRNAs between patients with and without distant metastasis. In total, 71 differentially expressed mRNAs were screened out with log_2_(fold change) > 1 or < -1 and *P* < 0.05. Significantly upregulated and downregulated mRNAs are shown as red and blue dots, respectively.

**Figure 3:**
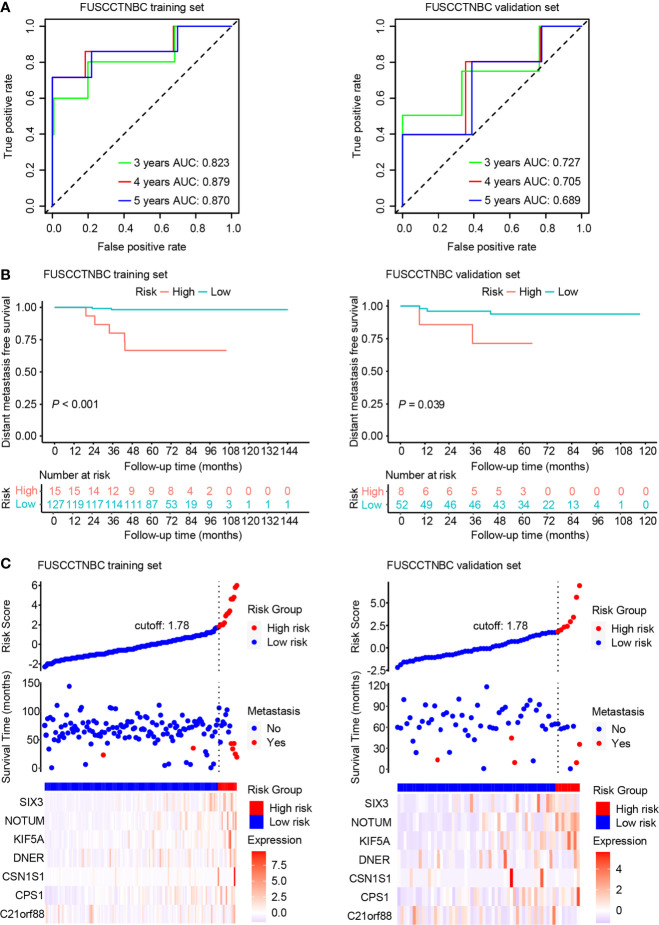
Time-dependent receiver operating characteristic (ROC), Kaplan–Meier survival analysis, and risk score analysis for the seven-gene signature in the training set and validation set of the lymph node-negative triple-negative breast cancer (TNBC) cohort. AUC, area under the curve. **(A)** Time-dependent ROC curves of the seven-gene signature for 3-, 4-, and 5-year distant metastasis-free survival (DMFS). **(B)** Kaplan–Meier plots of the seven-gene signature illustrating that the patients in the high-risk group showed poorer DMFS than those in the low-risk group. **(C)** Distribution of genomic risk score, DMFS status of patients, and heat map of seven differentially expressed mRNA expression profiles.

An overview of the study design is shown in **Figure 1**. Using log_2_(fold change) > 1 or < -1 and *P* < 0.05, we identified 71 differentially expressed mRNAs between the two groups with or without distant metastasis. We also performed Gene Set Enrichment Analysis of differentially expressed genes between the two groups with or without distant metastasis using the RNA-sequencing data. In patients with distant metastasis, 25 gene sets were significantly enriched at nominal *P* value < 0.05. The top ten gene sets enriched in 12 lymph node-negative TNBC patients with distant metastasis compared to 190 patients without distant metastasis were illustrated in **Figure S1**. In patients with distant metastasis, 56 mRNAs were upregulated, whereas 15 mRNAs were downregulated (**Figure 2**). We constructed a matrix integrating RNA-sequencing data of 71 differentially expressed mRNAs and clinicopathological data of all 202 patients. Next, patients were randomly classified into the training set (n = 142) and validation set (n = 60). There was no difference in all characteristics between the training and internal validation sets (**Table 1**). Seven genes, including *B3GALT5-AS1*, *DNER*, *CSN1S1*, *KIF5A*, *SIX3*, *NOTUM*, and *CPS1*, were selected using the LASSO Cox regression model in the training set. The summary of log_2_(fold change), multivariable Cox regression coefficient, hazard ratio, 95% confidence interval, and *P* value for selected genes are presented in **Table 2**. Time-dependent ROCs and Kaplan–Meier curves were used to evaluate the prognostic potential of the seven-gene signature for DMFS (**Figures 3A, B**). The AUC values for 3-, 4-, and 5-year DMFS were 0.823, 0.879, and 0.870 in the training set and 0.727, 0.705, and 0.689 in the validation set, respectively (**Figure 3A**). The formula of genomic risk score is as follows: genomic risk score = 0.18801037 × *DNER* + 0.28358112 × *CSN1S1* + 0.36011127 × *KIF5A* + 0.57677377 × *SIX3* + 0.70105693 × *NOTUM* + 0.74508978 × *CPS1* - 0.06761698 × *B3GALT5-AS1*. Patients were stratified into high- (n = 15) and low-risk groups (n = 127) by selecting the optimal cutoff value (1.78) in the training set (**Figures 3B, C**). Using the same cutoff value (1.78), the patients were also divided into high-risk (n = 8) and low-risk (n = 52) groups in the validation set (**Figures 3B, C**). The Kaplan-Meier analyses for DMFS as a function of the seven-gene signature showed highly significant differences between the high- and low-risk groups (**Figure 3B**, *P* < 0.001 in the training set; *P* = 0.039 in the validation set).

**Table 2:** Genes included in the seven-gene prognostic signature.

Gene symbol	Log_2_ FC^a^	Coefficient^b^	HR (95% CI)^b^	*P* ^b^
B3GALT5-AS1	1.18	-0.06761697	0.93 (0.41-2.15)	0.87
DNER	1.60	0.18801037	1.21 (0.39-3.73)	0.74
CSN1S1	1.61	0.28358112	1.33 (1.03-4.30)	0.11
KIF5A	1.10	0.36011127	1.43 (0.79-2.61)	0.24
SIX3	1.28	0.57677377	1.78 (0.92-3.44)	0.09
NOTUM	1.81	0.70105693	2.02 (1.22-3.33)	0.01
CPS1	1.51	0.74508978	2.11 (1.03-4.30)	0.04

FC, fold change; HR, hazard ratio; CI, confidence interval.

a The difference in the expression of seven genes between the group with and without distant metastasis was calculated using the limma package in R software.

b The coefficients, hazard ratios, 95% confidence intervals, and P values of seven genes were calculated using a multivariate Cox proportional hazards regression model.

### Construction and Validation of the Combined Gene and Clinical Model

**Figure 4:**
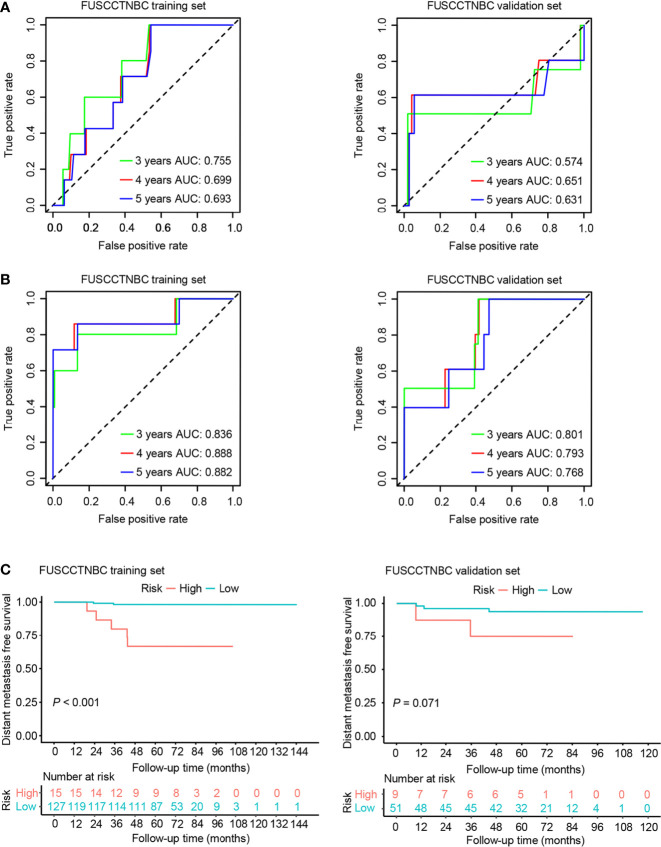
Time-dependent receiver operating characteristic (ROC) and Kaplan–Meier survival analysis for the clinical model and combined model in the training set and validation set of the lymph node-negative triple-negative breast cancer (TNBC) cohort. AUC, area under the curve. **(A)** Time-dependent ROC curves of the clinical model for 3-, 4-, and 5-year distant metastasis-free survival (DMFS). **(B)** Time-dependent ROC curves of the combined model for 3-, 4-, and 5-year DMFS. **(C)** Kaplan–Meier plots of the combined model illustrating that the patients in the high-risk group showed poorer DMFS than those in the low-risk group.

We also created a clinical prognostic model using the following clinically significant predictors: age and tumor size. The summary of multivariable Cox regression coefficient, hazard ratio, 95% confidence interval, and *P* value for age and tumor size are presented in **Table S1**. The formula of clinical risk score is as follows: clinical risk score = 0.21532 × Tumor size (cm) - 0.04466 × Age (years). The AUC values of the clinical model for 3-, 4-, and 5-year DMFS were 0.755, 0.699, and 0.693 in the training set and 0.574, 0.651, and 0.631 in the validation set, respectively (**Figure 4A**). The genomic risk score remained an independent prognostic factor in the multivariate Cox analysis after adjusting for patient age and tumor size in both the training set (hazard ratio = 2.64, 95% CI: 1.76-3.96, *P* < 0.001) and validation set (hazard ratio = 1.63, 95% CI: 1.07-2.49, *P* = 0.02). The combined risk score was derived from the genomic and clinical risk score as follows: combined risk score = 0.9702 × Genomic risk score + 1.0854 × Clinical risk score. After integrating the clinical model with the genomic risk score, the AUC values for 3-, 4-, and 5-year DMFS were 0.836, 0.888, and 0.882 in the training set, respectively (**Figure 4B**). The AUC values of the combined model remained high in the validation set with values of 0.801, 0.793, and 0.768 for 3-, 4-, and 5-year DMFS, respectively (**Figure 4B**). Patients were stratified into high- (n = 15 or 9) and low-risk groups (n = 127 or 51) in the training set or validation set (**Figure 4C**). The Kaplan-Meier analyses for DMFS as a function of the combined model showed a significant difference between the high- and low-risk groups in the training set (**Figure 4C**, *P* < 0.001). Likewise, the trend was also observed in the validation set (**Figure 4C**, *P* = 0.071).

### Construction and Validation of a Predictive Nomogram

**Figure 5:**
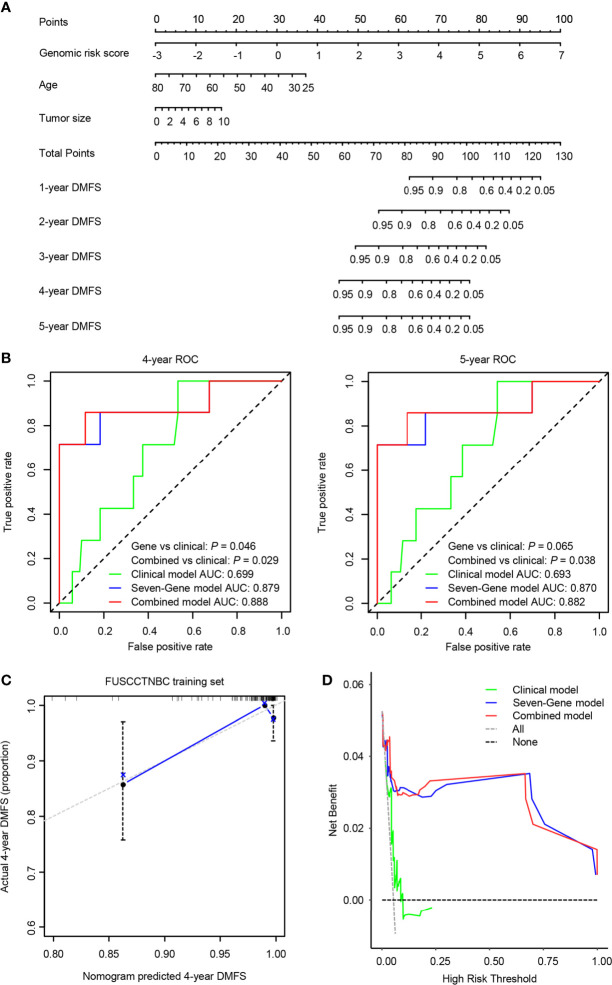
A predictive nomogram was established in the training set. AUC, area under the curve. **(A)** The nomogram was built by the seven-gene risk score and clinical characteristics, including age and tumor size. **(B)** The time-dependent receiver operating characteristic (ROC) curves of the seven-gene model, clinical model, and combined model for 4- and 5-year distant metastasis-free survival (DMFS). The combined model was better than the clinical model for predicting 4-year (*P* = 0.029) and 5-year (*P* = 0.038) DMFS. **(C)** Calibration plots of the nomogram for 4-year DMFS. **(D)** Decision curve analysis (DCA) of the seven-gene model, clinical model, and combined model for 4-year DMFS.

We integrated the seven-gene signature with age and tumor size to construct a prognostic nomogram in the training set (**Figure 5A**). The C-index value for the combined models was 0.874 in the training set and 0.805 in the validation set. The 4- and 5-year time-dependent ROC curves for the seven-gene, clinical, and combined models are illustrated in **Figure 5B**. Both the seven-gene model and combined model showed better prognostic performance than the clinical model for predicting 4-year DMFS (*P* = 0.046 for the gene model; *P* = 0.029 for the combined model). The combined model showed significantly better prognostic performance than the clinical model for predicting 5-year DMFS (*P* = 0.038), and the seven-gene model also trended toward significance (*P* = 0.065). The calibration analysis of the 4-year DMFS prediction is shown in **Figure 5C**. The solid blue line has a closer fit to the dotted gray line, indicating great predictive accuracy of the nomogram. Decision curve analysis (DCA) revealed that compared to the clinical model, the seven-gene model and combined model were superior in predicting 4-year DMFS (**Figure 5D**).

## Discussion

We constructed a novel seven-gene signature (*B3GALT5-AS1*, *DNER*, *CSN1S1*, *KIF5A*, *SIX3*, *NOTUM*, and *CPS1*) and a combined prognostic model integrating a seven-gene signature with patient age and tumor size to quantify the likelihood of distant metastasis in lymph node-negative TNBC. Both the seven-gene signature and the combined prognostic model had higher AUC values for 4- and 5-year survival than the clinical model. Patients were divided into low- and high-risk groups based on optimal cutoff values. Compared to the low-risk group, patients in the high-risk group had significantly poorer DMFS in both the training set and validation set. Finally, we constructed a prognostic nomogram and validated it in an internal validation set.

Several multigene assays have been employed in breast cancer, including the 76-gene signature, MammaPrint^®^ (70-gene profile), Breast Cancer Index (BCI) test, Oncotype^®^ DX Breast Recurrence Score (RS), EndoPredict^®^ (EP), and Prosigna^®^ (Risk Of Recurrence, ROR) (13, 28–32). None of the above is specifically designed and validated for TNBC patients. Most previous prognostic evaluation studies have focused on all TNBC patients (20, 33–37). One publication has reported the first validated proteomic signature of lymph node-negative TNBC patients (38), but all patients involved in this study were adjuvant treatment-naive, differing from actual clinical practice. The present study focused only on lymph node-negative TNBC patients with more than 90% of patients receiving adjuvant treatment. Apart from the study cohort, the flowchart to construct the gene signature in our study differed from previous studies. The seven differentially expressed mRNAs between the two groups with or without distant metastasis were utilized in our study, while we constructed our previous integrated mRNA-lncRNA signature after comparing the tumor tissues with the paired normal tissues as in most previous studies (39, 40). Therefore, genes selected for model development in the present study correlated more closely to prognosis based on well-documented follow-up information. Although more than 90% of patients received adjuvant chemotherapy in our cohort, the high-risk groups classified by the seven-gene signature and combined model presented poor DMFS within four years after surgery. Chemotherapy escalation may be required for these patients.

Among the seven genes, *B3GALT5-AS1* was the only RNA gene. A previous study has revealed the suppressive roles of the B3GALT5-AS1/miR-203/epithelial-mesenchymal transition (EMT) regulation axis in colon cancer liver metastasis (41). Similarly, *B3GALT5-AS1* was the only gene with a negative correlation coefficient in the present study. Delta/Notch-like EGF repeat containing (*DNER*) is a transmembrane protein that regulates EMT to enhance the proliferation and metastasis of breast cancer cells *via* the Wnt/β-catenin pathway (42). The other three genes, *SIX3*, *NOTUM*, and *CPS1*, have also been reported in other types of tumors. A systematic meta-analysis of non-small cell lung cancer has indicated that higher expression of SIX homeobox 3 (*SIX3*) is associated with a greater probability of tumorigenesis and a higher TNM stage (43). *NOTUM* acts as a key negative regulator of the Wnt signaling pathway, and knockdown of *NOTUM* genes inhibits the proliferation and migration of colorectal cancer cells (44). Previous studies have demonstrated that *CPS1* expression is upregulated in glioblastoma multiforme and that overexpression of *CPS1* is associated with poor therapeutic response and adverse outcomes among rectal cancer patients receiving concurrent chemoradiotherapy (45, 46). Inconsistent with our study, Mou et al. found a positive correlation between the lower expression of *CSN1S1* and patients surviving with breast cancer (47). Kinesin family member 5A (*KIF5A*) encodes a member of the kinesin family of proteins. Previous research has confirmed that kinesin overexpression correlates with specific taxane resistance in basal-like breast cancer cell lines and tissues (48). Investigational kinesin protein inhibitors, such as GSK-923295, may be promising drugs in the future.

Our study had several limitations. First, external validation is required to ensure generalization. Second, our study did not explore the expression and prognostic effects of the seven genes at the protein level due to the incomplete protein expression information of partial genes. Finally, the reliability of our prognostic model needs further clinical validation.

In conclusion, we identified and validated a novel seven-gene signature model and constructed a nomogram combined with the patient age and tumor size for predicting DMFS in lymph node-negative TNBC patients. A higher risk score may indicate an increased likelihood of distant metastasis and vice versa. After taking the potential benefits and increased risks of distant metastasis into account, treatment escalation may be considered as an alternative strategy for lymph node-negative TNBC patients with a high-risk score. In contrast, de-escalation chemotherapy might be taken into consideration in patients with a low-risk score.

## Data Availability Statement

The original contributions presented in the study are included in the article/**Supplementary Material**. Further inquiries can be directed to the corresponding authors.

## Ethics Statement

The studies involving human participants were reviewed and approved by Fudan University Shanghai Cancer Center. The patients/participants provided their written informed consent to participate in this study.

## Author Contributions

WP, CL, SJ, XJ, GD, and ZS: study concept and design. WP, CL, SJ, and GS: data analysis and interpretation. WP: wrote the first draft of the manuscript. WP, CL, and GS: visualization. XJ and ZS: funding acquisition. XJ, GD, and ZS: final approval. All authors contributed to the article and approved the submitted version.

## Funding

This study was funded by the National Natural Science Foundation of China (81902684) and Shanghai three year action plan for Traditional Chinese Medicine [ZY(2018-2020)-CCCX-2005-04]. The funders had no role in the design of the study and collection, analysis, interpretation of data or writing the manuscript.

## Conflict of Interest

The authors declare that the research was conducted in the absence of any commercial or financial relationships that could be construed as a potential conflict of interest.

## Publisher’s Note

All claims expressed in this article are solely those of the authors and do not necessarily represent those of their affiliated organizations, or those of the publisher, the editors and the reviewers. Any product that may be evaluated in this article, or claim that may be made by its manufacturer, is not guaranteed or endorsed by the publisher.
