# Supplementary material for: A Novel Seven Gene Signature-Based Prognostic Model to Predict Distant Metastasis of Lymph Node-Negative Triple-Negative Breast Cancer
Source: Front Oncol. 2021 Sep 16;11:746763. doi: 10.3389/fonc.2021.746763 (PMC8481824; doi:10.3389/fonc.2021.746763)
Supplement: Supplementary file 1 [file DataSheet_1.pdf]

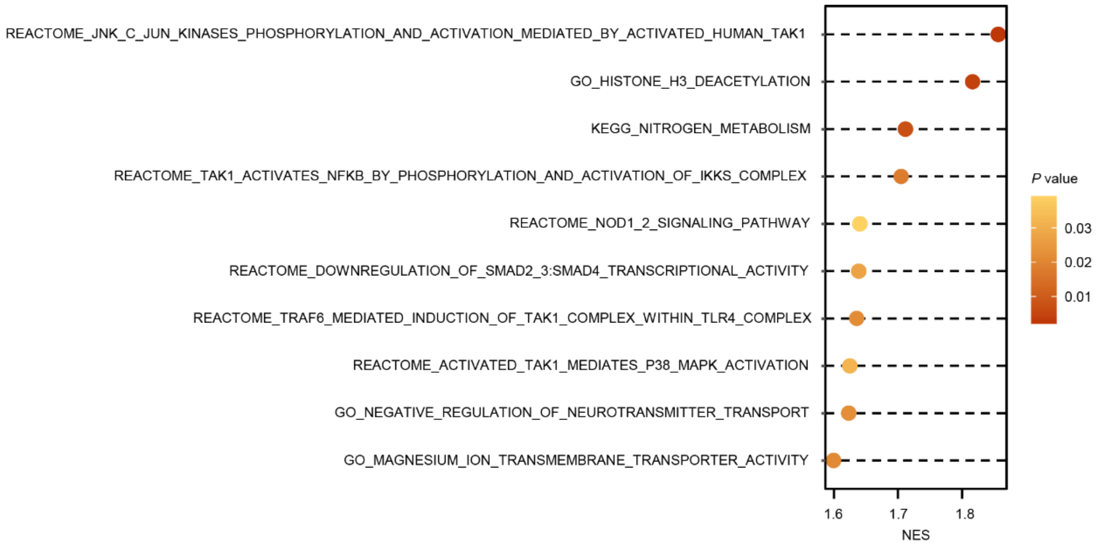


**Supplementary Figure Legend**

**Supplementary Figure 1.** The top ten gene sets enriched in 12 lymph node-negative triple-negative breast cancer patients with distant metastasis.

The top ten gene sets enriched in the subgroup with distant metastasis was shown ranking by the normalized enrichment score. Each enrichment is summarized as a bubble, darker as the enrichment was more significant.

NES, normalized enrichment score.
