# Supplementary material for: A Novel Seven Gene Signature-Based Prognostic Model to Predict Distant Metastasis of Lymph Node-Negative Triple-Negative Breast Cancer
Source: Front Oncol. 2021 Sep 16;11:746763. doi: 10.3389/fonc.2021.746763 (PMC8481824; doi:10.3389/fonc.2021.746763)
Supplement: Supplementary file 2 [file Table_1.docx]

**TABLE S1 Clinicopathological characteristics included in the clinical model.**

| **Clinical variables** | **Coefficient^a^** | **HR (95% CI)^a^** | ***P*^a^** |
| --- | --- | --- | --- |
| Age | -0.04466 | 0.96 (0.89-1.03) | 0.25 |
| Tumor size | 0.21532 | 1.24 (0.80-1.92) | 0.33 |

HR, hazard ratio; CI, confidence interval.

^a^The coefficients, hazard ratios, 95% confidence intervals, and *P* values of age and tumor size were calculated using a multivariate Cox proportional hazards regression model.
